# Appraisal of fungal infections during ECMO therapy

**DOI:** 10.1186/s13054-018-2082-1

**Published:** 2018-06-06

**Authors:** Nicolas Mongardon, Ophélie Constant, Fabio Silvio Taccone, Eric Levesque

**Affiliations:** 1 Service d’Anesthésie-Réanimation Chirurgicale, DHU A-TVB, Hôpitaux Universitaires Henri Mondor, Assistance Publique des Hôpitaux de Paris, CHU Henri Mondor, 51 avenue du Maréchal de Lattre de Tassigny, 94000 Créteil, France; 2 Faculté de Médecine, Université Paris Est, Créteil, France; 3 Inserm U955, Equipe 3, Stratégies Pharmacologiques et Thérapeutiques Expérimentales des Insuffisances cardiaques et coronaires, Créteil, France; 4 Department of Intensive Care, Clinique Universitaire de Bruxelles (CUB) Erasme, Université Libre de Bruxelles, Bruxelles, Belgique; 5 EA Dynamyc, UPEC ENVA, Créteil, France

We read with great interest the study of Cavayas and colleagues, which retrospectively investigated the occurrence and the impact of fungal infections in patients on extracorporeal membrane oxygenation (ECMO) included in the Extracorporeal Life Support Organization registry [1]. Despite the high selection of these patients, the authors concluded that fungal infections were not more frequent in patients on ECMO than in other critically ill patients but were independently associated with poor outcome. Because infectious complications are extremely important in ECMO patients [2], we would like to discuss some important issues raised by this study.

First, the epidemiology, definitions, and respective weights of the different fungal infections should be clearly distinguished. The diagnosis of invasive fungal infection and, particularly, invasive pulmonary aspergillosis (IPA) is problematic in critically ill patients. In the study by Cavayas et al. the lack of data (i.e., host characteristics, clinical features, mycological criteria) on patients with *Aspergillus*-positive culture precludes a definitive diagnosis in most cases. Similarly, no distinction is proposed between colonization and proven or putative IPA, which is possible using the clinical algorithm adapted for critically ill patients who lack the usual host factors, such as those with hematological cancer or on prolonged immunosuppressive therapies [3]. Consequently, the outcome of patients with IPA and *Aspergillus* colonization is extremely different.

Second, *Aspergillus* and *Candida* diseases are different fungal infections, whereas the characteristics of patients with these two diseases were summarized in one single cohort in this study. The authors have logically shown how risk factors for *Aspergillus* (underlying immunodeficiency or initial lung injury, i.e., hematological malignancies, influenza infection, solid organ transplantation) are significantly different from those for *Candida* infections (non-specific severity surrogates, i.e., older age, overweight, sepsis, and renal replacement therapy). Moreover, recent reports have emphasized the presence of underlying “immunoparalysis” related to critical illness and the role of severe lung injury on venous-venous (VV)-ECMO for the occurrence of IPA [4, 5]; these data were unfortunately not available in this registry. Also, invasive candidiasis is likely to develop because of exposure to broad-spectrum antibiotics, multiple line cannulation, parenteral nutrition, or multiple surgical procedures; the absence of these variables in the multivariable analysis significantly limits the interpretation of the main findings in this study. Epidemiological interpretations are further hampered by the lack of data on delay of occurrence of these infections.

Finally, VV-ECMO and veno-arterial (VA)-ECMO were analyzed as a whole. However, VV-ECMO supports the most severe forms of respiratory failure; pulmonary damage increases susceptibility to respiratory pathogens, like *Aspergillus*, in this setting. On the contrary, VA-ECMO supports refractory cardiogenic shock with multi-organ failure and may be at higher risk for *Candida* infections. Thus, a separated analysis according to the type of ECMO support might have been more informative on the role of fungal infection in ECMO patients.

In light of these supplementary concerns and the retrospective design, the initial conclusions of this study should be interpreted cautiously. These data call for further studies and actions to better understand and recognize secondary infections in patients in whom ECMO is a life-saving support treatment.
